# PSIONplus: Accurate Sequence-Based Predictor of Ion Channels and Their Types

**DOI:** 10.1371/journal.pone.0152964

**Published:** 2016-04-04

**Authors:** Jianzhao Gao, Wei Cui, Yajun Sheng, Jishou Ruan, Lukasz Kurgan

**Affiliations:** 1 School of Mathematical Sciences and LPMC, Nankai University, Tianjin, People's Republic of China; 2 Department of Statistics, University of California Riverside, Riverside, California, United States of America; 3 Graduate School at Shenzhen, Tsinghua University, Shenzhen, People's Republic of China; 4 State Key Laboratory of Medicinal Chemical Biology, Nankai University, Tianjin, People’s Republic of China; 5 Department of Electrical and Computer Engineering, University of Alberta, Edmonton, Alberta, Canada; 6 Department of Computer Science, Virginia Commonwealth University, Richmond, Virginia, United States of America; Zhejiang University, CHINA

## Abstract

Ion channels are a class of membrane proteins that attracts a significant amount of basic research, also being potential drug targets. High-throughput identification of these channels is hampered by the low levels of availability of their structures and an observation that use of sequence similarity offers limited predictive quality. Consequently, several machine learning predictors of ion channels from protein sequences that do not rely on high sequence similarity were developed. However, only one of these methods offers a wide scope by predicting ion channels, their types and four major subtypes of the voltage-gated channels. Moreover, this and other existing predictors utilize relatively simple predictive models that limit their accuracy. We propose a novel and accurate predictor of ion channels, their types and the four subtypes of the voltage-gated channels called PSIONplus. Our method combines a support vector machine model and a sequence similarity search with BLAST. The originality of PSIONplus stems from the use of a more sophisticated machine learning model that for the first time in this area utilizes evolutionary profiles and predicted secondary structure, solvent accessibility and intrinsic disorder. We empirically demonstrate that the evolutionary profiles provide the strongest predictive input among new and previously used input types. We also show that all new types of inputs contribute to the prediction. Results on an independent test dataset reveal that PSIONplus obtains relatively good predictive performance and outperforms existing methods. It secures accuracies of 85.4% and 68.3% for the prediction of ion channels and their types, respectively, and the average accuracy of 96.4% for the discrimination of the four ion channel subtypes. Standalone version of PSIONplus is freely available from https://sourceforge.net/projects/psion/

## Introduction

Ion channels are membrane proteins that facilitate the flow of ions through the lipid membranes [1, 2]. Besides their biological importance, they are of substantial research interest in the context of drug development [3–5]. There are over 300 types of ion channels in living cells [6]. They differ in their structures and cellular functions. Ion channels are gated by variety of factors including voltage, ligands, membrane tension, temperature and light [7]. Considering their mechanism of activation, ion channels are mainly classified into the voltage-gated and ligand-gated ion channels [8, 9]. The ligand-gated ion channels open and close depending on the interactions with specific ligands while the voltage-gated ion channels function in response to the voltage gradient across the membrane. The voltage-gated ion channels can be further classified into several subtypes including potassium (K), sodium (Na), calcium (Ca), anion ion channels, proton channels, transient receptor potential channels and hyperpolarization-activated cyclic nucleotide-gated channels [9].

Studies of structure and function of ion channels continue to attract significant research attention [10–16]. As a highlight, recent years have seen strong interest in the role of ion channels as antiviral targets [17]. In the specific case of influenza A, the structure and mechanistic details of the voltage-gated M2 proton channel was recently analyzed [18–20] and a few high-profile articles on the potential therapy that targets this channel were published [21, 22]. The strong research interest and ubiquity of ion channels [23–25] motivate the development of methods that predict them from protein sequences.

A naïve approach that finds ion channels based on their sequence similarity to sequences of known channels was found to be flawed [26]. Consequently, more sophisticated, machine learning methods which can predict different types and subtypes of ion channels that are dissimilar in their sequences were developed. In one of the first attempts, Liu *et al*. [27] proposed a method to predict voltage-gated potassium channels and certain families of this subtype of channels based on a simple dipeptide compositions extracted from an input sequence and Support Vector Machine (SVM) predictive model. Using a more advanced design that included SVM model and dipeptide composition combined with PSI-BLAST-based [28] and HMMER-based [29] similarity searches, Saha *et al*. [30] have developed the VGIchan method that predicts voltage-gated ion channels and their subtypes. More recently, in 2011 Lin *et al*. [31] proposed a method that offers a much wider scope including the prediction of ion channels, ion channels types, and the four subtypes of the voltage-gated ion channels. However, the design of this method was similar to the method by Liu *et al*. [27] and involved the use of a subset of amino acid and dipeptide composition values and the SVM model. In 2012, Chen and Lin [32] published a narrower in scope approach that predicts subfamilies of the voltage-gated potassium channels, yet again using a similar design that applies SVM and amino acid and dipeptide composition. Finally, in 2014 the same group released a slightly improved method for the prediction of subfamilies of the voltage-gated potassium channels that applies an empirically selected subset of tripeptide composition values and the SVM model [33]. All but one of the existing methods are characterized by a relatively narrow scope being restricted to either voltage-gated potassium channels or voltage-gated ion channels. The one method that was developed in 2011 by Lin *et al*. offers a comprehensive scope but utilizes a relatively simple design that is similar to all other methods. Our aim is to provide a novel method that provides similarly comprehensive scope, i.e., it predicts whether a given sequence is an ion channel, what type of the channel it is, and which subtype of the voltage-gated ion channel it is, while utilizing a more advanced design that should lead to an improved predictive performance. Our method considers an empirically selected collection of inputs that for the first time in this area utilizes physiochemical properties of amino acid derived from the input protein chain, position specific scoring matrix (PSSM) profiles generated by PSI-BLAST, and predicted secondary structure, relative solvent accessibility and intrinsic disorder.

## Materials and Methods

### Datasets

The data used to build the proposed prediction method are taken from Lin *et al*. [31]. Protein sequences were downloaded from UniProt [34] and the Ligand-Gated Ion channel database (http://www.ebi.ac.uk/compneur-srv/LGICdb/LGICdb.php) [35]. The chains that include non-standard amino acid types, fragments of proteins, and proteins annotated based on homology or predictions were excluded. The remaining sequences were clustered at 40% identity using CD-HIT [36] to remove similar chains. This resulted in 298 ion channel proteins with 150 ligand-gated and 148 voltage-gated ion channels. The voltage-gated ion channels include 81 potassium (K), 29 calcium (Ca), 12 sodium (Na) and 26 voltage-gated anion channels. To facilitate assessment of prediction of the ion-channels vs. non-ion channel dataset, 300 membrane proteins that were randomly selected from UniProt and that share <40% identity to the ion channel proteins were designated as the non-ion channel proteins. These data were used to derive three training datasets (Table 1). TRAIN_ION_ is used to develop predictor that discriminates the ion channel and non-ion channel chains. TRAIN_VLG_ is used to build predictor of ion channel types, i.e., voltage-gated vs. and ligand-gated ion channel. Finally, TRAIN_VGS_ is the training dataset for prediction of the four subtypes of the voltage-gated ion channels.

**Table 1:** Datasets used to design and test the proposed method.

Dataset name	Annotations	Number of chains
TRAIN_ION_	Ion channel	298
	Non-ion channel	300
TRAIN_VLG_	Voltage-gated channel	148
	Ligand-gated channel	150
TRAIN_VGS_	Potassium(K)	81
	Calcium(Ca)	29
	Sodium(Na)	12
	Anion	26
TEST30_ION_	Ion channel	94
	Non-ion channel	104
TEST30_VLG_	Voltage-gated channel	43
	Ligand-gated channel	17
TEST60_VGS_	Potassium(K)	120
	Calcium(Ca)	49
	Sodium(Na)	23
	Anion	47

We also developed three new test datasets that include proteins that are dissimilar to proteins in the three training datasets. These test datasets, which were not used to design our predictor, were collected from UniProt two years after the dataset from Lin *et al*. was established. We followed the protocol from ref. [31]. We collected reviewed chains annotated with the following five Gene Ontology keywords: 1) “ligand-gated channel”; 2) “voltage-gated” and “potassium channel”; 3)“voltage-gated” and “calcium channel”; 4) “voltage-gated” and “sodium channel”; and 5) “voltage-gated” and “anion channel”. Next, we excluded annotations that were inferred from homology, which are predicted and uncertain. The non-ion channel proteins were randomly selected from the UniProt to match the number of the ion-channels. We excluded chains with non-standard amino acid types (X, B and U) and chains that have similarity of over 30% with the proteins in any of the training datasets, based on the clustering with CD-HIT. Consequently, the TEST30_ION_ and TEST30_VLG_ datasets, which are used to assess prediction of ion channels and ion channel types, include 198 and 60 proteins, respectively (Table 1). Using the 30% similarity cutoff did not allow us to collect a sufficient number of proteins for the four subtypes of the voltage-gated ion channels to perform tests. Thus, the test set for these subtypes, TEST60_VGS_, is based on 60% similarity threshold to the training proteins and includes total of 239 proteins (Table 1).

### Assessment of the predictive performance

The predictors of the ion channels, their types and subtypes generate either a binary outcome (ion channel vs. non-ion channel and voltage-gated vs. ligand-gated) or four outcomes (potassium, sodium, calcium and anion ion channel). The assessment of these predictions uses the same measures as in the related works, including accuracy [27, 30–33] and Matthews correlation coefficient (MCC) [27, 30, 32, 33]:

Accuracy=(TP+TN)/(TP+FP+TN+FN) (1)

$$MCC=\left(TP*TN+FP*FN\right)/\sqrt{\left(TP+FP)(TP+FN)(TN+FP)(TN+FN\right)}$$ (2)

where *TP* is true positive, *TN* is true negative, *FP* is false positive, and *FN* is false negative. We also compute F_measure_, which is a weighted average of the precision and recall and has maximal and minimal values of 1 and 0, respectively:

$$F_{measure}=2TP/(2TP+FN+FP)=2*precision*recall/\left(precision+recall\right)$$ (3)

The accuracy, MCC and F_measure_ are computed for the two binary predictions and for each of the four outcomes in the prediction of voltage-gated ion subtypes. We also compute average accuracy, MCC, F_measure_ and Q_4_ accuracy to summarize the overall prediction over the four subtypes:

$$Q_{4}=\sum_{i=1..n}(TP_{i}/N)$$ (4)

where N is total number of sequences and *n* = 4 is number of classes.

The entire design process, which includes feature selection and parameterization of the predictive model, was run using five-fold cross validation on the training datasets; the same features and parameters are used in all benchmark tests. The resulting design is compared using *N*-fold cross-validation (jackknife test) on the training datasets with the results in ref. [31] where the same jackknife test was performed. Finally, we computed predictive performance on the test datasets utilizing our model trained on the corresponding training datasets.

### Overall architecture of the predictor

The proposed method, PSIONplus (Predictor from Sequence of ION channels plus BLAST) combines predictions from a machine learning model (PSION) and from sequence alignment with BLAST. PSION consist of three modules: (1) PSION_ION_ model that predicts whether a given input sequence is an ion channel; (2) PSION_VLG_ model that predicts whether a given ion channel is voltage- or ligand-gated; and (3) PSION_VGS_ model that generates predictions of the four subtypes of the voltage-gated channels. The three models share common architecture where the input protein sequences is first processed to obtain its evolutionary profile and predicted secondary structure (SS), relative solvent accessibility (RSA), and intrinsic disorder (ID). Next, this information is combined with the sequence itself to generate a set of numeric features which are input into a predictive model. We applied SVM to generate the model given its widespread use in the prediction of ion channels [27, 30–33] and results from ref. [31] that empirically demonstrate that this machine learning model is superior when compared to four other classifiers including Naïve Bayes, RBF network, logistic regression and random forest. We used the LIBSVM implementation of SVM [37]. The model outputs a prediction based on the numeric scores generated by SVM (ion channel vs. non-ion channel, voltage-gated vs. ligand-gated channel, one subtype of voltage-gated channels). LIBSVM [37] uses “one-against-one” approach for the multi-class classification of the subtypes. LIBSVM constructs k*(*k*-1)/2 binary classifiers to develop predictor for *k* classes. In our case, for *k* = 4 it constructs 6 binary classifiers. For binary classification, LIBSVM estimates the probabilities for each class using parametric sigmoid function as described in ref. [38]. The output class is the class with the higher probability. For the multi-class classification, LIBSVM collects all pairwise class probabilities that are estimated as in ref. [38], generates one probability for each class based on an optimization described in refs. [39],[40], and outputs the class with the highest probability. We designed the SVM model by considering a large pool of features, performing empirical selection of a subset of relevant and well-performing features, and empirically parameterizing the predictive model.

### Considered input features

We considered seven groups of features which are based on (1) amino acid composition of the input sequence; (2) dipeptide composition of the input sequence; (3) physiochemical properties of the amino acid in the input sequence; (4) predicted SS; (5) predicted RSA; (6) predicted ID; and (7) PSSM profile.

The amino acid composition is defined as the number of residues of a given amino acid type divided by the sequence length. This type of features was used by the prior methods [31, 32]. The dipeptide composition is the composition of all 400 pairs of amino acid types and it was also used in the related works [27, 30–32]. The physiochemical properties are a feature type that is new to this area. We considered hydrophilicity [41], hydrophobicity [42], polarity [43], flexibility [44], propensity for beta-turns [45] and transfer free energy [46], which are quantified based on the corresponding amino acid indices from the AAindex database [47]. The selection is motivated by the fact that the same properties have been used in similar works [48, 49]. We computed the average and standard deviation for each of the six properties over all residues in the input sequence.

We also utilized new features that are based on several structural properties that were predicted from the input chain. SS and ID are predicted by the standalone version v3.3 of PSIPRED [50] and v2.43 of DISOPRED [51], respectively. RSA is predicted with SPINEX [52] and is defined as the ratio of solvent accessible surface area of a residue observed in its three dimensional structure to that observed in an extended Ala-X-Ala tripeptide conformation [53, 54]. The PSSM profiles have been widely used in various related predictive efforts [55–61]. We used the *blastpgp* implementation of PSI-BLAST with the default three iterations (-*j* 3) utilizing the *nr* protein database to calculate the PSSM profiles for the input protein sequence.

**Fig 1:**
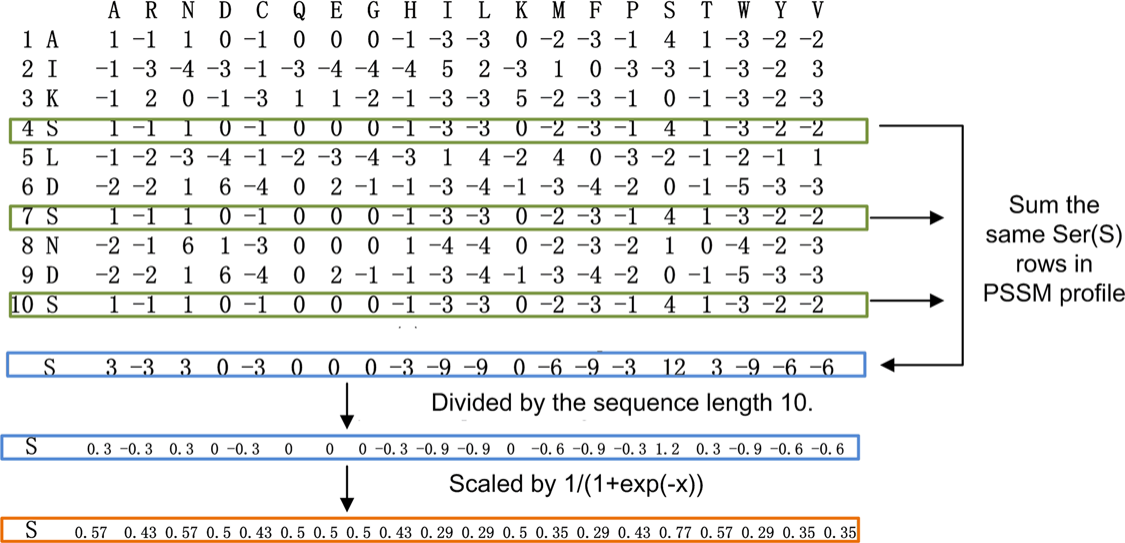
Example computation of scores from the PSSM profile.

Altogether, we considered the following 878 features:

- *AA_j*, composition of *j* = 1, 2,.., 20 amino acid (AA) types (20 features)
- *Dipeptide_{AA}_{AA}*, the composition of AA pairs (20*20 = 400 features).
- *AAproperty*_*i*_*_{avg*, *sd}*, the average (*avg*) or standard deviation (*sd*) of *i* = {1 for hydrophilicity, 2 for hydrophobicity, 3 for polarity, 4 for flexibility, 5 for beta-turns, 6 for transfer free energy} amino acid property over all AA in the input protein chain. These features quantify average and variability of propensity for a given property over the entire input protein (6*2 = 12 features)
- *Num_SS_Seg*, the total number of predicted secondary structure segments in the input protein chain (1 feature)
- *Num_{C*,*H*,*E}_Seg*, the number of predicted coil, helix, or strands segments in the input protein chain (3 features)
- *CV_{C*,*H*,*E}*_*Seg*_*_{min*, *max}*, the minimal and maximal length of the predicted coil, helix, strand segments divided by the protein length (3*2 = 6 features)
- *Composition_{C*, *H*, *E}*, the composition of coil, helix, or strand residues, i.e., the number of coil, helix, or strand residues divided by the sequence length (3 features)
- *Total_DisNonDis_Seg*, the total number of predicted disordered and structured (non-disordered) segments in the input protein chain (1 feature)
- *Num_{Dis*, *NonDis}*_*Seg*_, the number of disorder and structured (non-disordered) segments in the input protein chain (2 features)
- *CV_{Dis*, *NonDis}*_*Seg*_*_{min*, *max}*, the minimal and maximal length of disorder and structured (non-disordered) segments divided by the sequence length (2*2 = 4 features)
- *Composition_{Dis*, *NonDis}*, the composition of disorder and structured residues, i.e., the number of disorder and structured residues divided by the sequence length (2 features)
- *{Bd*, *Ed}_{0*.*25*, *0*.*75}*, the composition of buried and exposed residues, i.e., the number of buried and exposed residues divided by the sequence length. A given residue is considered to be buried if it’s predicted RSA < 0.25 or 0.75; otherwise, it is assumed to be exposed. These features quantify to some degree the overall shape of the input protein (2*2 = 4 features)
- *RSA*_*{min*, *max}*_*_Seg{4*,*6*,*8*,*10*,*12*,*14*,*16*,*18*,*20*,*22}*, the minimal or maximal value of the average of the predicted RSA values for segments which are at least 4, 6, 8, 10, 12, 14, 16, 18, 20, or 22 residues long. These features identify long segments of either exposed or buried residues, which again is related to the shape of the protein molecule (10*2 = 20 features)
- *PSSM_{AA*_*1*_*}_{AA*_*2*_*}*, PSSM profile scores where AA_1_ and AA_2_ stand one of the 20 amino acid types in the input protein chain and in the columns of the PSSM profile, respectively. These features quantify evolutionary conservation of individual amino acid types in the input protein chain. We compute the PSSM profile scores by summing up rows in the PSSM profiles for the same AA type. Next, each element in the resulting 400 dimensional vector (20 amino acid types * 20 columns in the PSSM profile) is divided by the length of the sequence and normalized by1/(1+exp(-x)). Example is shown in Fig 1. A similar PSSM profile score was used to classify transporters [62] (20*20 = 400 features)

Each feature was normalized into [–1, 1] interval based on the min-max normalization: (2*x—x_*min*_*—x*_*max*_) / (*x*_*max*_*—x*_*min*_*)* where *x* is a value of a given feature *X* and *x*_*min*_ and *x*_*max*_ are the minimal and maximal values of *X*, respectively.

### Feature selection and optimization of the predictive model

Given that some of the considered features may not be useful for the prediction of the ion channels and some of the features could be correlated with each other (redundant), we performed empirical selection of a subset of predictive and non-redundant features. The selection was based on the biserial correlation coefficients (BCC) computed between values of a given feature and the binary outcomes; this correlation was also used in related studies [63, 64]. We performed selection for each of the three types of outcomes, i.e., prediction of ion channels, ion channel types, and subtypes of voltage-gated channels. First, a given training dataset was randomly divided into the five training and test folds to implement the five-fold cross validation protocol. We ranked the features according their average BCC over the five training folds. Second, we removed features that are characterized by low predictive power by considering five cut-offs = {0.1, 0.15, 0.2, 0.25 and 0.3}, i.e., features with the average BCC below a given cut-off were excluded. In the third step we removed correlated features. We selected the feature with the highest average BCC and added the next ranked feature into the selected set of features if the Pearson’s correlation coefficient (PCC) of this feature with every feature in the selected feature set was below a given cut-off value = {0.7, 0.75, 0.8, 0.85 and 0.9}. The use of the two cut-offs results in 5*5 = 25 feature sets. In the fourth step, we further reduced the number of features in each of the 25 feature sets using wrapper-based feature selection. This type of feature selection scores a given feature set based on predictive quality of a prediction model that uses this feature set. We quantified predictive quality with MCC based on predictions using the five-fold cross validation protocol on the corresponding training dataset using the SVM classifier and chose the feature sets that gives the highest MCC score. To clarify, in our cross-validation the training dataset was randomly partitioned into five equally sized subsets. One subset was used as a test dataset and the remaining four subsets were used as a training dataset. This was repeated five times, each time choosing a different subset as the test dataset and using same features and parameters of the prediction model. We combined predictions from the five test subsets together to produce a single MCC value (Table 2) and we also averaged the five MCCs from the 5 test subsets. (Table A in S1 File). In the wrapper selection we attempted to remove each of the features in the set, measured the MCC of the smaller set, and accepted this removal in case if the MCC value increases. As an alternative approach, in the fourth step we implemented feature selection with the principal components analysis (PCA) using SVM classifier and 5-fold cross validation on the training dataset. We considered nine values of the cut-off on the variance value that is covered by the PCA = {0.1, 0.2,…, 0.9} to generate the corresponding nine feature sets. Next, like in the wrapper selection we considered removing one PCA-based feature at the time and we removed it only if this increases MCC. Finally, in the fifth step for each resulting reduced feature set we optimized parameters of the SVM model. Following the Lin *et al*. [31] we used the radial basis function (RBF) kernel and performed grid search over the regularization parameter *C* = 2^−2^, 2^−1^,…, 2^4^ and width of the RBF kernel *gamma* = 2^−11^, 2^−10^,…,2^0^. We selected the set of parameters that provides the highest value of MCC in the five-fold cross validation on the corresponding training dataset. The results are summarized in Table 2 and Table B in S1 File.

**Table 2:** Results of the feature selection and optimization of the three predictive models for ion channels, ion channel types, and subtypes of voltage-gated channels.

BCC	PCC	Maximal MCC over selected feature sets (step 4)			Optimal SVM parameters (*C*, *gamma*)			Number of features		
		Ion channel	Ion channel type	Voltage-gated channel subtype	Ion channel	Ion channel type	Voltage-gated channel subtype	Ion channel	Ion channel type	Voltage-gated channel subtype
0.1	0.9	0.835	0.927	0.697	8, 0.0625	4, 0.0625	16, 0.0625	190	158	46
	0.85	0.832	0.934	0.664	8, 0.0625	4, 0.03125	8, 0.25	205	122	29
	0.8	0.830	0.921	0.656	16, 0.03125	0.5, 0.0625	4, 0.0625	171	102	48
	0.75	**0.836**	0.934	0.665	**4, 0.03125**	2, 0.0625	16,0.015625	**172**	103	71
	0.7	0.796	0.933	0.614	8, 0.0625	4, 0.0625	16, 0.007812	150	107	63
0.15	0.9	0.798	0.928	0.668	2, 0.125	2, 0.125	4, 0.0625	138	109	53
	0.85	0.788	0.934	0.664	4, 0.125	2, 0.0625	8, 0.25	134	102	29
	0.8	0.777	0.927	0.656	4, 0.125	2, 0.0625	4, 0.0625	92	80	48
	0.75	0.802	0.907	0.665	4, 0.125	4, 0.125	16,0.015625	114	110	71
	0.7	0.787	0.922	0.614	2, 0.0625	0.5, 0.03125	16, 0.007812	99	82	63
0.2	0.9	0.773	0.920	0.715	8, 0.03125	1, 0.125	4, 0.0625	70	77	48
	0.85	0.766	0.908	0.562	8, 0.125	4, 0.125	2, 0.25	69	94	37
	0.8	0.769	0.914	0.619	8, 0.125	0.5, 0.25	16, 0.0625	72	76	68
	0.75	0.763	**0.934**	0.618	8, 0.03125	**2, 0.25**	4, 0.25	60	**56**	28
	0.7	0.776	0.920	0.641	16, 0.125	1, 0.0625	2, 0.25	64	65	32
0.25	0.9	0.743	0.921	0.695	4, 0.25	1, 0.25	16, 0.0625	40	63	32
	0.85	0.756	0.893	0.670	8, 0.25	16, 0.015625	16, 0.0625	38	60	33
	0.8	0.760	0.913	0.682	4, 0.5	2, 0.125	16, 0.25	39	69	26
	0.75	0.759	0.893	**0.735**	8, 0.5	0.5, 0.25	**4, 0.125**	29	41	**25**
	0.7	0.741	0.880	0.589	2, 0.5	1, 0.25	8, 0.125	27	42	26
0.3	0.9	0.686	0.908	0.574	2, 0.5	1, 0.25	16, 0.0625	22	53	31
	0.85	0.700	0.907	0.634	1, 1	2, 0.125	16, 0.25	21	37	25
	0.8	0.700	0.914	0.716	1, 1	1, 0.5	8, 0.25	21	38	31
	0.75	0.700	0.907	0.653	1, 1	1, 0.5	16, 0.125	20	33	25
	0.7	0.675	0.893	0.573	0.5, 1.0	2, 0.015625	8, 0.5	16	33	22
Cutoff on variance in PCA		Maximal MCC over selected feature sets (step 4)			Optimal SVM parameters (*C*, *gamma*)			Number of features		
		Ion channel	Ion channel type	Voltage-gated channel subtype	Ion channel	Ion channel type	Voltage-gated channel subtype	Ion channel	Ion channel type	Voltage-gated channel subtype
0.1		0.445	0.582	0.168	8, 0.00977	16, 0.001953	8, 0.125000	2	1	1
0.2		0.670	0.582	0.240	4,0.007812	16,0.001953	16,0.12500	4	1	1
0.3		0.670	0.817	0.397	4,0.007812	1,0.015625	32,0.007812	4	5	2
0.4		0.680	0.776	0.486	2,0.03125	1,0.015625	8,0.000488	7	6	6
0.5		0.719	0.850	0.503	16,0.003906	2,0.015625	2,0.015625	13	14	6
0.6		0.803	0.870	0.505	4,0.003906	2,0.007812	4,0.007812	32	21	6
0.7		0.767	0.896	0.669	4,0.001953	4,0.003906	4,0.003906	66	38	26
0.8		0.804	0.935	0.661	8,0.001953	16,0.000977	2,0.007812	116	69	22
0.9		0.810	0.922	0.596	8,0.000977	2,0.001953	8,0.007812	153	65	30

The table shows results for different cut-offs for the minimal biserial correlation coefficients (BCC) computed between values of a given feature and the binary outcomes (step 2 of feature selection) and the maximal Pearson’s correlation coefficient (PCC) between features (step 3), the maximal MCC value obtained via wrapper-based feature selection (step 4) and the optimal SVM parameters (step 5) that were computed via five-fold cross validation on the corresponding training dataset, and the final number of selected features. The lower part of the table shows results for an alternative feature selection based on Principal Component Analysis (PCA) with different cut-off on the value of variance. Predictions from the five test folds in the cross validations were combined together to produce a single MCC value. The selected setup for each of the three predictors is shown in bold font.

For the prediction of ion-channels, the correlation-based feature selection results in the predictor that secures MCC = 0.836 which is higher than MCC = 0.810 that was obtained with the PCA-based approach. For the ion channel type model, both feature selection lead to models with similar predictive quality (MCC = 0.934 and 0.935) while the correlation-based approach uses fewer features (56 vs. 69). For the prediction of the voltage-gated channel subtypes, correlation- and PCA-based feature selections correspond to MCC = 0.735 and 0.669, respectively (Table 2). We note that results obtained by averaging the MCC over the five cross validation folds lead to consistent results with the same optimal designs that secure MCC = 0.836±0.051 for prediction of ion-channels, MCC = 0.933±0.041 for the ion channel type, and MCC = 0.740±0.100 for the voltage-gated channel subtypes (Table B in S1 File). Consequently, the PSIONplus predictor is built utilizing the feature sets generated with the correlation-based feature selection, which are shown in bold font in Table 2 and Table B in S1 File. The predictor of ion channels, PSION_ION_, uses 172 features and SVM with *C* = 4 and *gamma* = 0.03125; predictor for ion channel types, PSION_VLG_, utilizes 56 features and SVM with *C* = 2 and *gamma =* 0. 25; and for voltage-gated ion channel subtypes, PSION_VGS_, we apply 25 features and SVM with *C* = 4 and *gamma* = 0.125.

### PSIONplus: combination of SVM model and BLAST

PSIONplus is implemented by combining the prediction of the selected SVM model and sequence alignment computed with BLAST against a dataset of annotated proteins. To compute the prediction from BLAST, we query a given test protein sequence against the sequences from the training dataset and transfer annotation from the most similar hit given that it is sufficiently similar. We only use training sequences for which the corresponding *e*-value is better than a threshold that we establish based on cross validation on the training datasets. We performed grid search over the following set of *e*-values: 10^−6^, 10^−5^,…,10^0^, 10^1^. We selected the values that provide the highest MCC in the five-fold cross validation on a given training set. Consequently, PSIONplus uses *e*-value = 0.001 for the prediction of ion channels (based on the TRAIN_ION_ dataset), *e*-value = 10 for the ion channel types (based on the TRAIN_VLG_ dataset), and *e*-value = 0.001 for the voltage-gated channel subtypes (based on the TRAIN_VGS_ dataset).

**Fig 2:**
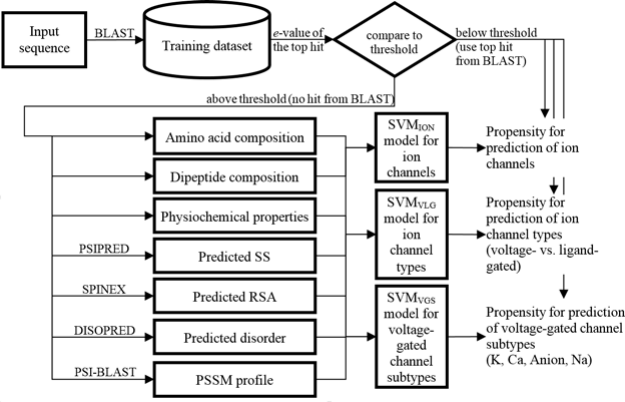
Workflow of the PSIONplus model. SS: secondary structure, RSA: relative solvent accessibility.

Besides the binary prediction, the numeric score generated by BLAST equals to normalized *e*-value of the first hit: *score* = *threshold*/(*threshold* + *e*-value); this way the score is higher when similarity is higher, which is when the *e*-value is smaller. If there is no hit from BLAST (all *e*-values > threshold) then PSIONplus uses the score from the SVM model. Otherwise, PSIONplus uses the score from BLAST. Fig 2 shows the workflow of PSIONplus.

## Results

### Analysis of predictive model

Table 3 summarizes the selected features for each of the three SVM models: SVM_ION_ for the prediction of ion channels, SVM_VLG_ for the prediction of ion channel types, and SVM_VGS_ for the prediction of voltage-gated channel subtypes. It reveals that majority of these features are based on amino acid pairs and PSSM profile scores. However, all types of features were selected in at least one predictive model. This demonstrates that the new types of features that we introduce including PSSM profiles, predicted SS, ID and RSA and physiochemical properties of AAs, contribute to the predictive performance.

**Table 3:** Summary of considered and selected features used by the PSION predictor.

Feature group	Number of features	Number of selected features		
		SVM_ION_	SVM_VLG_	SVM_VGS_
PSSM profile scores	400	75	29	18
Dipeptide composition	400	82	24	4
Predicted relative solvent accessibility	24	4	0	0
Amino acid composition	20	5	1	0
Predicted secondary structures	13	2	1	1
Properties of amino acid	12	3	1	0
Predicted intrinsic disorder	9	1	0	2
Total	878	172	56	25

To quantify relative impact of each type of features we divided the selected features into five groups that are based on dipeptide composition, predicted intrinsic disorder, predicted relative solvent accessibility, predicted secondary structure, and PSSM-based profiles. Next, using features from a given group, we optimized SVM model based on the five-fold cross validation on the corresponding training dataset using the same procedure as described in Materials and Methods section. The accuracies obtained by each feature group on each of the three training datasets are shown in Table 4; we note that in some cases the results are not available if none of the features from a given group was used in the corresponding model. We computed a single value of accuracy based the results that are combined over all test folds (entire test datasets). The best performing feature group is based on the PSSM profiles, which we introduced into the prediction of the ion channels and their types. However, each of the remaining feature groups also obtains relative strong accuracy. For the prediction of the ion channels the lowest accuracy is 60.3% while a baseline classifier, which would always predict the most frequent outcome, has accuracy of 100%*(300/598) = 50.1% (Table 1). Similarly, for the prediction of ion channel type and voltage-gated channel subtype the lowest accuracies are 68.1% and 62.2% compared to the baseline accuracies of 100%*(150/298) = 50.3% and 100%*(81/148) = 54.7%, respectively. Most importantly, the PSION model that combines all these features obtains higher predictive performance compared with the best performing feature group. By using all features together the error rates are reduced by 100%*(91.6–89.6)/(100–89.6) = 19.2% for the prediction of ion channels, by 100%*(96.3–95.6)/(100–95.6) = 15.9% for the prediction of ion channel types, and by 100%*(88.5–81.8)/(100–81.8) = 36.8% for the prediction of voltage-gates channel subtypes (Table 4). This suggests that aggregation of the various types of previously used and new feature types leads to an improved predictive performance.

**Table 4:** Accuracy obtained based on the cross validation on the training datasets TRAIN_ION_ and TRAIN_VLG_ and Q_4_ based on the cross validation on the TRAIN_VGS_ dataset by different groups of input features.

Models	TRAIN_ION_ (accuracy)	TRAIN_VLG_ (accuracy)	TRAIN_VGS_ (Q_4_)
Model based on the PSSM profile	89.6	95.6	81.8
Model based on the dipeptide composition	84.5	87.6	65.5
Model based on the predicted relative solvent accessibility	79.8	not used	not used
Model based on the predicted secondary structure	69.9	68.1	62.2
Model based on the predicted intrinsic disorder	60.3	not used	62.2
Model based on all features	91.6	96.3	88.5

We computed a single value of accuracy based the results that are combined over all test folds (entire test datasets)

### Comparative analysis of results on the training datasets

Table 5 compares results generated by PSIONplus and its two modules based on SVM and BLAST based on the jackknife tests on the training datasets with the equivalent results on the same datasets from the only other method that also predicts ion channels, their types, and subtypes of voltage-gated channels from ref. [31]. We compared the accuracies and number of features since the MCC and F_measure_ values were not provided in the other article; these measures are compared on the test datasets.

**Table 5:** Summary of results based on the jackknife and 5-fold cross validation (5-cv) tests on the training datasets TRAIN_ION_, TRAIN_VLG_ and TRAIN_VGS_.

Evaluation measure	Method	TRAIN_ION_	TRAIN_VLG_	TRAIN_VGS_					
		Ion-channel vs. non-ion channel	Voltage-gated vs. ligand-gated	Potassium	Anion	Calcium	Sodium	Q_4_	Average of the four subtypes
*Accuracy*	Lin *et al*.	86.6	92.6	92.6	84.6	82.8	75.0	87.8	83.8
*(Jackknife)*	SVM model	91.5	96.3	93.9	97.3	91.9	96.6	89.9	94.9
	BLAST	98.0	99.7	98.6	99.3	98.0	98.6	97.3	98.6
	PSIONplus	97.7	100	99.3	100	98.0	98.6	98.0	99.0
*MCC*	SVM model	0.830	0.927	0.880	0.905	0.732	0.782	NA	0.825
*(Jackknife)*	BLAST	0.960	0.993	0.973	0.977	0.935	0.909	NA	0.948
	PSIONplus	0.953	1	0.986	1	0.935	0.909	NA	0.958
*MCC*	SVM model	0.833	0.934	0.736	0.855	0.441	0.695	NA	0.682
*(5-cv)*	BLAST	0.944	0.980	0.774	0.831	0.597	0.773	NA	0.744
	PSIONplus	0.940	0.993	0.846	0.929	0.650	0.773	NA	0.799
*Sensitivity*	SVM model	93.0	98.0	98.8	84.6	72.4	83.3	NA	84.8
*(Jackknife)*	BLAST	97.0	99.3	100	96.2	93.1	91.7	NA	95.2
	PSIONplus	98.7	100	100	100	93.1	91.7	NA	96.2
*Sensitivity*	SVM model	90.3	98.6	96.3	80.8	41.4	75.0	NA	73.4
*(5-cv)*	BLAST	95.0	98.6	100	73.1	44.8	91.7	NA	77.4
	PSIONplus	97.7	100	100	88.5	51.7	91.7	NA	83.0
*# of features*	Lin *et al*.	140	159	104	104	104	104	NA	NA
	PSION	172	56	25	25	25	25	NA	NA

Results of PSIONplus and its two modules based on SVM and BLAST are compared with the method by Lin *et al*. MCC and F_measure_ were not reported in the article by Lin *et al*. and thus only accuracy is compared. The best accuracy values for each dataset is shown in bold. For the cross-validation tests we computed a single value of accuracy, MCC and sensitivity based in the results that are combined over all test folds (entire test datasets). NA means “not applicable”.

The accuracy of the SVM model used in the PSIONplus predictor is higher than the accuracy of the method by Lin *et al*. across all three types of predictions. The corresponding error rates of our SVM are reduced by 100%*(91.5–86.6)/(100–86.6) = 36.6%, 100%*(96.3–92.6)/(100–92.6) = 50%, and 100%*(89.9–87.8)/(100–87.8) = 17.2% for the prediction of ion channels, ion channel types, and voltage-gates channel subtypes, respectively. Since our predictor uses a similar or smaller number of features and predictive model compared to the other method, the improved predictive performance stems from the use of novel feature types. Moreover, the PSIONplus that combines this SVM model with sequence alignment obtains even better predictive quality. The corresponding error rates of are reduced by 100%*(97.7–86.6)/(100–86.6) = 82.8%, 100%*(100–92.6)/(100–92.6) = 100%, and 100%*(98–87.8)/(100–87.8) = 83.6% when compared with method by Lin *et al*.

We compared the predictive performance of PSIONplus and BLAST on the training datasets. In Table 5, PSIONplus achieves accuracies of 97.7 and 100 and Q_4_ of 97.3 on the TRAIN_ION_, TRAIN_VLG_ and TRAIN_VGS_ datasets based on the jackknife test. BLAST achieves comparable levels of accuracy at 98.0, 99.7, and 98.0_,_ respectively. Similar conclusion is true when measuring predictive quality with MCC and both cross-validation and jackknife tests. The strong performance of BLAST is due to the relatively high sequence similarity in these training datasets. Moreover, we also compared sensitivity (defined as the fraction of correctly predicted true positives) of PSIONplus and BLAST. We note that PSIONplus achieves higher sensitivity values at 98.7 and 100 (97.7 and 100) on TRAIN_ION_ and TRAIN_VLG_, and higher average sensitivity at 96.2 (83.0) on TRAIN_VGS_ compared to 97.0, 99.3 and 95.2 (95.0, 98.6, and 77.4) of BLAST when using jackknife (cross-validation) test. These differences indicate that PSIONplus that combines BLAST with the SVM can identify more positives than BLAST alone. This means that some of the correct predictions generated by PSIONplus come from the SVM model.

### Comparative analysis of results on the test datasets

The predictive quality of PSIONplus is compared using the test datasets with the method by Lin *et al*. [31] and with alignment with BLAST for the prediction of ion channels, their types, and subtypes of the voltage-gated channels, and with VGIchan [30] for the prediction of ion channels (Table 6). The empirical results suggest that PSIONplus offers substantially higher predictive quality than VGIchan with MCC equal 0.71 vs. 0.49. Comparison with the predictor by Lin *et al*. leads to similar conclusions to the conclusions drawn based on the results on the training datasets. PSIONplus obtains higher values of MCC by 0.08 and 0.29, accuracy by 4.6 and 5.0 and F_measure_ by 3.7 and 1.0 for the prediction of ion channels and ion channel types, respectively. The results concerning the prediction of the voltage-gated channel subtypes are similar, with the differences in average F_measure_, average MCC and average accuracy equal to 7.4, 1.1 and 4.0, respectively. The Q_4_ of PSIONplus is 92.9 on TEST60_VGS,_ which is higher than the Q_4_ of 84.9 from Lin et *al*.

**Table 6:** Summary of results on the test datasets TEST30_ION_, TEST30_VLG_, and TEST60_VGS_.

Dataset	Prediction outcome	Method	F_measure_	MCC	Accuracy	Q_4_
TEST30_ION_	Ion-channel vs. non-ion channel	VGIchan	63.0	0.49	72.7	NA
		Lin *et al*. 2011	81.7	0.63	80.8	NA
		BLAST	64.3	0.56	74.7	NA
		PSIONplus	**85.4**	**0.71**	**85.4**	NA
		Confidence interval of PSIONplus	86.0(±3.7)	0.73(±0.07)	86.3(±3.3)	NA
TEST30_VLG_	Voltage-gated vs. ligand-gated	Lin *et al*. 2011	76.6	-0.06	63.3	NA
		BLAST	**77.6**	**0.23**	**68.3**	NA
		PSIONplus	**77.6**	**0.23**	**68.3**	NA
		Confidence interval of PSIONplus	78.1(±6.1)	0.22(±0.15)	68.7(±7.4)	NA
TEST60_VGS_	Potassium	Lin *et al*. 2011	87.6	0.74	86.6	NA
		BLAST	91.6	0.83	90.8	NA
		PSIONplus	**94.8**	**0.90**	**94.6**	NA
	Anion	Lin *et al*. 2011	86.7	0.85	95.4	NA
		BLAST	86.7	0.85	95.4	NA
		PSIONplus	**88.1**	**0.87**	**95.8**	NA
	Calcium	Lin *et al*. 2011	73.7	0.67	89.5	NA
		BLAST	91.1	**0.90**	**96.7**	NA
		PSIONplus	**92.0**	**0.90**	**96.7**	NA
	Sodium	Lin *et al*. 2011	90.5	0.90	98.3	NA
		BLAST	**93.0**	**0.93**	**98.7**	NA
		PSIONplus	**93.0**	**0.93**	**98.7**	NA
	Average over	Lin *et al*. 2011	84.6	0.79	92.4	84.9
	all subtypes	BLAST	90.6	0.88	95.4	90.8
		PSIONplus	**92.0**	**0.90**	**96.4**	**92.9**
		Confidence interval of PSIONplus	91.9(±2.1)	0.90(±0.03)	96.4(±0.9)	92.9(±1.7)

Results of PSIONplus are compared with VGIchan on the TEST30_VLG_ dataset, and with the method by Lin *et al*. and BLAST on all datasets. Best MCC, F_measure_ and accuracy values for each dataset are shown in bold. Confidence intervals are obtained by computing average and standard deviations (shown in brackets) of 10 repetition of the test where in each repetition we randomly select 50% of test data set. NA means “not applicable”; for the two-class classification the Q_4_ equals accuracy.

We compared PSIONplus with BLAST on the three test datasets in Table 6. PSIONplus achieves better accuracy = 85.4 than the accuracy = 74.7 by BLAST on the TEST30_ION_ dataset and the same accuracy on the TEST30_VLG_ dataset. For the prediction of voltage-gated four subtypes, PSIONplus obtain average accuracy = 96.4 and MCC = 0.90 which is higher than the average accuracy = 95.4 and MCC = 0.88 by BLAST. The Q_4_ of PSIONplus is 92.9 which is again higher than the Q_4_ of BLAST at 90.8. This shows that PSIONplus improves over the sequence alignment and justifies the use of the SVM model in the PSIONplus.

We also computed confidence intervals for PSIONplus. We randomly selected 50% of the test proteins and calculated the corresponding F_measure_s, MCCs and accuracies. This was repeated 10 times and we computed the averages and standard deviations over these 10 repetitions. Table 6 shows that the standard deviations are relatively low on the TEST30_ION_ and TEST60_VGS_ datasets. The standard deviations are larger on the TEST30_VLG_ dataset, however, the results obtained by the method by Lin *et al*. are also proportionally lower.

Finally, we estimated false positive rate, defined as the number of false positives divided by the number of actual negatives, of PSIONplus. Our method achieves the false positive rate = 19.2%, 52.9%, and 3.2% on the TEST30_ION_, TEST30_VLG_ and TEST60_VGS_ datasets, respectively (see Table B in S1 File), compared to 1%, 52.9% and 4.6% by BLAST_._ Although the false positive rate of PSIONplus is higher than for BLAST on TEST30_ION_, the sensitivity (true positive rate) of PSIONplus = 90.4% and is much higher than BLAST’s sensitivity that is 47.9% (see Table B in S1 File). This means that the increase by 42.5% in sensitivity by PSIONplus is traded for the higher by 18.2% false positive rate. However, for the TEST60_VGS_ dataset PSIONplus secures both lower average false positive rate and higher average sensitivity when compared to BLAST.

## Discussion

We propose the PSIONplus method for accurate prediction of ion channels proteins and their types, and subtypes of the voltage-gated ion channels. Empirical results show that combination of results generated by SVM model with the alignment by BLAST that is implemented in PSIONplus leads to improved predictive performance for the prediction of ion channels and voltage-gated channel subtypes when compared to using just BLAST. Results on the benchmark datasets that are independent of the datasets used to design our predictor reveal that PSIONplus obtains relatively good predictive performance. Its accuracy is 85.4% for the prediction of ion channels, 68.3% for the prediction of ion channel types, and its average accuracy is 96.4% for the prediction of the four subtypes of the voltage-gated channels. PSIONplus outperforms existing methods for the prediction of ion channels including VGIchan and the method by Lin *et al*.

PSIONplus is the first method that uses new types of predictive inputs including PSSM profiles and predicted secondary structure, solvent accessibility and intrinsic disorder. We note that computation of the PSSM profiles and structural predictions is relatively computationally-heavy and it may take up to several minutes for a single sequence on a desktop computer. However, our empirical tests demonstrate that the PSSM profiles provide the strongest predictive input and that all new types of features contribute to the prediction, i.e., prediction using the combined set of all inputs is better when compared to using individual sets of features, and every feature set individually provides good predictive quality. Given the strong predictive value of the PSSM profiles, one interesting extension of our method would be to develop features based on generic (instead of position specific like PSSM) sequence similarity utilizing for instance the BLOSUM matrices. Another potentially impactful extension would be to apply an alternative methods to generate alignment profiles, such as HHBLITS[65] that was shown to be competitive with the currently used PSI-BLAST.

Standalone version of PSION can be freely downloaded from https://sourceforge.net/projects/psion/.

## Supporting Information

S1 File This file includes Tables A and B. (PDF)

## References

[1] Domene C, Haider S, Sansom MS. Ion channel structures: a review of recent progress. Current opinion in drug discovery & development. 2003;6(5):611–9. . 14579510

[2] Doyle DA. Molecular insights into ion channel function—(Review). Mol Membr Biol. 2004;21(4):221–5. 10.1080/09687680410001716844 . 15371011

[3] Camerino DC, Tricarico D, Desaphy JF. Ion channel pharmacology. Neurotherapeutics: the journal of the American Society for Experimental NeuroTherapeutics. 2007;4(2):184–98. Epub 2007/03/31. 10.1016/j.nurt.2007.01.013 . 17395128

[4] Camerino DC, Desaphy JF, Tricarico D, Pierno S, Liantonio A. Therapeutic approaches to ion channel diseases. Advances in genetics. 2008;64:81–145. Epub 2009/01/24. 10.1016/S0065-2660(08)00804-3 . 19161833

[5] Verkman AS, Galietta LJ. Chloride channels as drug targets. Nature reviews Drug discovery. 2009;8(2):153–71. Epub 2009/01/21. 10.1038/nrd2780 . 19153558 PMC3601949

[6] Gabashvili IS, Sokolowski BH, Morton CC, Giersch AB. Ion channel gene expression in the inner ear. Journal of the Association for Research in Otolaryngology: JARO. 2007;8(3):305–28. Epub 2007/06/02. 10.1007/s10162-007-0082-y 17541769 PMC2538437

[7] Banghart MR, Volgraf M, Trauner D. Engineering light-gated ion channels. Biochemistry. 2006;45(51):15129–41. 10.1021/bi0618058 . 17176035

[8] Ger MF, Rendon G, Tilson JL, Jakobsson E. Domain-based identification and analysis of glutamate receptor ion channels and their relatives in prokaryotes. PloS one. 2010;5(10):e12827 Epub 2010/10/16. 10.1371/journal.pone.0012827 20949136 PMC2950845

[9] Tabassum N, Feroz A. Ion Channels and their Modulation. Journal of Applied Pharmaceutical Science. 2011;01(01):6.

[10] Catterall WA. Ion channel voltage sensors: structure, function, and pathophysiology. Neuron. 2010;67(6):915–28. 10.1016/j.neuron.2010.08.021 20869590 PMC2950829

[11] Chou KC. Insights from modeling three-dimensional structures of the human potassium and sodium channels. Journal of proteome research. 2004;3(4):856–61. Epub 2004/09/14. . 15359741 10.1021/pr049931q

[12] Corry B. Understanding ion channel selectivity and gating and their role in cellular signalling. Mol Biosyst. 2006;2(11):527–35. 10.1039/B610062g . 17216034

[13] Konijnenberg A, Yilmaz D, Ingolfsson HI, Dimitrova A, Marrink SJ, Li ZL, et al Global structural changes of an ion channel during its gating are followed by ion mobility mass spectrometry. P Natl Acad Sci USA. 2014;111(48):17170–5. 10.1073/pnas.1413118111 . PMC4260606 25404294

[14] Doyle DA. Structural changes during ion channel gating. Trends Neurosci. 2004;27(6):298–302. 10.1016/j.tins.2004.04.004 . 15165732

[15] Tillman TS, Cascio M. Effects of membrane lipids on ion channel structure and function. Cell Biochem Biophys. 2003;38(2):161–90. 10.1385/Cbb:38:2:161 . 12777713

[16] Chung SH, Kuyucak S. Recent advances in ion channel research. Bba-Biomembranes. 2002;1565(2):267–86. doi: Pii S0005-2736(02)00574-6 10.1016/S0005-2736(02)00574-6 . 12409200

[17] Liang X, Li ZY. Ion channels as antivirus targets. Virologica Sinica. 2010;25(4):267–80. 10.1007/s12250-010-3136-y . 20960300 PMC8227895

[18] Huang RB, Du QS, Wang CH, Chou KC. An in-depth analysis of the biological functional studies based on the NMR M2 channel structure of influenza A virus. Biochemical and biophysical research communications. 2008;377(4):1243–7. Epub 2008/11/11. 10.1016/j.bbrc.2008.10.148 . 18996090

[19] Schnell JR, Chou JJ. Structure and mechanism of the M2 proton channel of influenza A virus. Nature. 2008;451(7178):591–5. Epub 2008/02/01. 10.1038/nature06531 18235503 PMC3108054

[20] Hu F, Luo W, Hong M. Mechanisms of proton conduction and gating in influenza M2 proton channels from solid-state NMR. Science. 2010;330(6003):505–8. 10.1126/science.1191714 20966251 PMC4102303

[21] Cady SD, Schmidt-Rohr K, Wang J, Soto CS, Degrado WF, Hong M. Structure of the amantadine binding site of influenza M2 proton channels in lipid bilayers. Nature. 2010;463(7281):689–92. 10.1038/nature08722 20130653 PMC2818718

[22] Wang J, Wu Y, Ma C, Fiorin G, Wang J, Pinto LH, et al Structure and inhibition of the drug-resistant S31N mutant of the M2 ion channel of influenza A virus. Proc Natl Acad Sci U S A. 2013;110(4):1315–20. 10.1073/pnas.1216526110 23302696 PMC3557100

[23] Le Novere N, Changeux JP. LGICdb: the ligand-gated ion channel database. Nucleic acids research. 2001;29(1):294–5. 11125117 10.1093/nar/29.1.294 PMC29772

[24] Jegla TJ, Zmasek CM, Batalov S, Nayak SK. Evolution of the human ion channel set. Combinatorial chemistry & high throughput screening. 2009;12(1):2–23. . 19149488 10.2174/138620709787047957

[25] Gallin WJ, Boutet PA. VKCDB: voltage-gated K+ channel database updated and upgraded. Nucleic acids research. 2011;39(Database issue):D362–6. 10.1093/nar/gkq1000 20972209 PMC3013635

[26] Fodor AA, Aldrich RW. Statistical limits to the identification of ion channel domains by sequence similarity. The Journal of general physiology. 2006;127(6):755–66. 10.1085/jgp.200509419 16735758 PMC2151544

[27] Liu LX, Li ML, Tan FY, Lu MC, Wang KL, Guo YZ, et al Local sequence information-based support vector machine to classify voltage-gated potassium channels. Acta biochimica et biophysica Sinica. 2006;38(6):363–71. Epub 2006/06/09. . 16761093 10.1111/j.1745-7270.2006.00177.x

[28] Altschul SF, Madden TL, Schaffer AA, Zhang J, Zhang Z, Miller W, et al Gapped BLAST and PSI-BLAST: a new generation of protein database search programs. Nucleic Acids Res. 1997;25(17):3389–402. 9254694 10.1093/nar/25.17.3389 PMC146917

[29] Eddy SR. Profile hidden Markov models. Bioinformatics. 1998;14(9):755–63. . 9918945 10.1093/bioinformatics/14.9.755

[30] Saha S, Zack J, Singh B, Raghava GP. VGIchan: prediction and classification of voltage-gated ion channels. Genomics, proteomics & bioinformatics. 2006;4(4):253–8. Epub 2007/05/29. 10.1016/S1672-0229(07)60006-0 . 17531801 PMC5054079

[31] Lin H, Ding H. Predicting ion channels and their types by the dipeptide mode of pseudo amino acid composition. Journal of theoretical biology. 2011;269(1):64–9. Epub 2010/10/26. 10.1016/j.jtbi.2010.10.019 . 20969879

[32] Chen W, Lin H. Identification of voltage-gated potassium channel subfamilies from sequence information using support vector machine. Comput Biol Med. 2012;42(4):504–7. 10.1016/j.compbiomed.2012.01.003 . 22297432

[33] Liu WX, Deng EZ, Chen W, Lin H. Identifying the Subfamilies of Voltage-Gated Potassium Channels Using Feature Selection Technique. Int J Mol Sci. 2014;15(7):12940–51. 10.3390/ijms150712940 . 25054318 PMC4139883

[34] Consortium TU. Reorganizing the protein space at the Universal Protein Resource (UniProt). Nucleic acids research. 2012;40(Database issue) Epub 2011 Nov 18. 10.1093/nar/gkr981 PMC3245120 22102590

[35] Donizelli M, Djite MA, Le Novere N. LGICdb: a manually curated sequence database after the genomes. Nucleic acids research. 2006;34(Database issue):D267–9. Epub 2005/12/31. 10.1093/nar/gkj104 16381861 PMC1347466

[36] Li W, Godzik A. Cd-hit: a fast program for clustering and comparing large sets of protein or nucleotide sequences. Bioinformatics. 2006;22(13):1658–9. 10.1093/bioinformatics/btl158 . 16731699

[37] Chang C-C, Lin C-J. LIBSVM: a library for support vector machines. ACM Transactions on Intelligent Systems and Technology. 2011;2(3):27:1-:.

[38] Platt JC. Probabilistic outputs for support vector machines and comparison to regularized likelihood methods In: Smola J. BP, Scholkopf B., Schuurmans D., editor. Advances in Large Margin Classifiers. Cambridge: MIT Press; 2000 p. 61–73.

[39] Wu TF, Lin CJ, Weng RC. Probability estimates for multi-class classification by pairwise coupling. Journal of Machine Learning Research. 2004;5:31.

[40] Lin H-T, Lin CJ, Weng R.C. A note on Platt's probabilistic outputs for support vector machines. Machine Learning. 2007;68:10.

[41] Kuhn LA, Swanson CA, Pique ME, Tainer JA, Getzoff ED. Atomic and residue hydrophilicity in the context of folded protein structures. Proteins. 1995;23:536–47. 8749849 10.1002/prot.340230408

[42] Eisenberg D. Three-dimensional structure of membrane and surface proteins. Ann Rev Biochem. 1984;53:595–623. 6383201 10.1146/annurev.bi.53.070184.003115

[43] Grantham R. Amino acid difference formula to help explain protein evolution. Science. 1974;185:862–4. 4843792 10.1126/science.185.4154.862

[44] Vihinen M, Torkkila E, Riikonen P. Accuracy of protein flexibility predictions. Proteins. 1994;19:141–9. 8090708 10.1002/prot.340190207

[45] Chou PY, Fasman GD. Empirical predictions of protein conformation. Ann Rev Biochem. 1978;47:251–76. 354496 10.1146/annurev.bi.47.070178.001343

[46] Janin J. Surface and inside volumes in globular proteins. Nature. 1979;277:491–2. 763335 10.1038/277491a0

[47] Kawashima S, Pokarowski P, Pokarowska M, Kolinski A, Katayama T, Kanehisa M. AAindex: amino acid index database, progress report 2008. Nucleic acids research. 2008;36(Database issue):D202–5. 10.1093/nar/gkm998 17998252 PMC2238890

[48] Rubinstein ND, Mayrose I, Martz E, Pupko T. Epitopia: a web-server for predicting B-cell epitopes. BMC Bioinformatics. 2009;10:287 10.1186/1471-2105-10-287 19751513 PMC2751785

[49] Zhang W, Niu Y, Xiong Y, Zhao M, Yu R, Liu J. Computational prediction of conformational B-cell epitopes from antigen primary structures by ensemble learning. PloS one. 2012;7(8):e43575 Epub 2012/08/29. 10.1371/journal.pone.0043575 22927994 PMC3424238

[50] Jones DT. Protein secondary structure prediction based on position-specific scoring matrices. Journal of molecular biology. 1999;292(2):195–202. 10.1006/jmbi.1999.3091 . 10493868

[51] Ward JJ, Sodhi JS, McGuffin LJ, Buxton BF, Jones DT. Prediction and functional analysis of native disorder in proteins from the three kingdoms of life. Journal of molecular biology. 2004;337(3):635–45. Epub 2004/03/17. 10.1016/j.jmb.2004.02.002 . 15019783

[52] Faraggi E, Zhang T, Yang Y, Kurgan L, Zhou Y. SPINE X: improving protein secondary structure prediction by multistep learning coupled with prediction of solvent accessible surface area and backbone torsion angles. Journal of computational chemistry. 2012;33(3):259–67. Epub 2011/11/03. 10.1002/jcc.21968 22045506 PMC3240697

[53] Ahmad S, Gromiha MM, Sarai A. Real value prediction of solvent accessibility from amino acid sequence. Proteins. 2003;50(4):629–35. Epub 2003/02/11. 10.1002/prot.10328 . 12577269

[54] Ahmad S, Gromiha MM, Sarai A. Analysis and prediction of DNA-binding proteins and their binding residues based on composition, sequence and structural information. Bioinformatics. 2004;20(4):477–86. 10.1093/bioinformatics/btg432 . 14990443

[55] Xie D, Li A, Wang M, Fan Z, Feng H. LOCSVMPSI: a web server for subcellular localization of eukaryotic proteins using SVM and profile of PSI-BLAST. Nucleic acids research. 2005;33(Web Server issue):W105–10. Epub 2005/06/28. 10.1093/nar/gki359 15980436 PMC1160120

[56] Ou YY, Gromiha MM, Chen SA, Suwa M. TMBETADISC-RBF: Discrimination of beta-barrel membrane proteins using RBF networks and PSSM profiles. Computational biology and chemistry. 2008;32(3):227–31. Epub 2008/04/25. 10.1016/j.compbiolchem.2008.03.002 . 18434251

[57] Kumar M, Gromiha MM, Raghava GPS. SVM based prediction of RNA-binding proteins using binding residues and evolutionary information. J Mol Recognit. 2011;24(2):303–13. 10.1002/Jmr.1061 . 20677174

[58] Chen K, Mizianty MJ, Kurgan L. ATPsite: sequence-based prediction of ATP-binding residues. Proteome Sci. 2011;9. doi: Artn S4 10.1186/1477-5956-9-S1-S4 . PMC3289083 22165846

[59] Zheng C, Kurgan L. Prediction of beta-turns at over 80% accuracy based on an ensemble of predicted secondary structures and multiple alignments. BMC bioinformatics. 2008;9. doi: Artn 430 10.1186/1471-2105-9-430 . PMC2613158 18847492

[60] Zhang H, Zhang T, Chen K, Shen SY, Ruan JS, Kurgan L. Sequence based residue depth prediction using evolutionary information and predicted secondary structure. BMC bioinformatics. 2008;9. doi: Artn 388 10.1186/1471-2105-9-388 . PMC2567998 18803867

[61] Yan RX, Xu D, Yang JY, Walker S, Zhang Y. A comparative assessment and analysis of 20 representative sequence alignment methods for protein structure prediction. Sci Rep-Uk. 2013;3. doi: Artn 2619 10.1038/Srep02619 . PMC3965362 24018415

[62] Ou YY, Chen SA, Gromiha MM. Classification of transporters using efficient radial basis function networks with position-specific scoring matrices and biochemical properties. Proteins. 2010;78(7):1789–97. Epub 2010/03/03. 10.1002/prot.22694 . 20196081

[63] Gao J, Faraggi E, Zhou Y, Ruan J, Kurgan L. BEST: improved prediction of B-cell epitopes from antigen sequences. PloS one. 2012;7(6):e40104 10.1371/journal.pone.0040104 22761950 PMC3384636

[64] Mizianty MJ, Kurgan L. Sequence-based prediction of protein crystallization, purification and production propensity. Bioinformatics. 2011;27(13):i24–33. Epub 2011/06/21. 10.1093/bioinformatics/btr229 21685077 PMC3117383

[65] Remmert M, Biegert A, Hauser A, Söding J. HHblits: lightning-fast iterative protein sequence searching by HMM-HMM alignment. Nat Methods. 2012;9:3. 22198341 10.1038/nmeth.1818

