# Supplementary material for: PSIONplus: Accurate Sequence-Based Predictor of Ion Channels and Their Types
Source: PLoS One. 2016 Apr 4;11(4):e0152964. doi: 10.1371/journal.pone.0152964 (PMC4820270; doi:10.1371/journal.pone.0152964)
Supplement: S1 File — (PDF) [file pone.0152964.s001.pdf]

Supporting Information File 1

PSIONplus: accurate sequence-based predictor of ion channels and their types

Jianzhao Gao[1], Wei Cui[2], Yajun Sheng[3], Jishou Ruan[1,4*] and Lukasz Kurgan[5,6*]

[1]School of Mathematical Sciences and LPMC, Nankai University, Tianjin, People's Republic of China.

[2]Department of Statistics, University of California, Riverside, California, United States.

[3]Graduate School at Shenzhen, Tsinghua University, Shenzhen, People's Republic of China.

[4]State Key Laboratory of Medicinal Chemical Biology, Nankai University, Tianjin, People's Republic of China.

[5]Department of Electrical and Computer Engineering, University of Alberta, Edmonton, Alberta, Canada.

[6]Department of Computer Science, Virginia Commonwealth University, Richmond, Virginia, USA.

* Correspondence to:
Lukasz Kurgan, email: lkurgan@vcu.edu
Jishou Ruan, email: jsruan@nankai.edu.cn

Table A. Results of the feature selection and optimization of the three predictive models for ion channels, ion channel types, and subtypes of voltage-gated channels.

The table shows results for different cut-offs for the minimal biserial correlation coefficients (BCC) computed between values of a given feature and the binary outcomes (step 2 of feature selection) and the maximal Pearson's correlation coefficient (PCC) between features (step 3), the maximal MCC value obtained via wrapper-based feature selection (step 4) and the optimal SVM parameters (step 5) that were computed via five-fold cross validation on the corresponding training dataset, and the final number of selected features. Predictions from the five test folds in the cross validations were averaged and we also show the corresponding standard deviations in round brackets. The selected setup for each of the three predictors is shown in bold font.

BCC	PCC	Maximal value of average (over 5 folds) MCC based on selected feature sets (step 4)			Optimal SVM parameters (±*C*, *gamma*)			Number of features		
		Ion channel	Ion channel type	Voltage-gated channel subtype	Ion channel	Ion channel type	Voltage-gated channel subtype	Ion channel	Ion channel type	Voltage-gated channel subtype
0.1	0.9	0.835 (±0.053)	0.927 (±0.055)	0.684 (±0.152)	8, 0.0625	4, 0.0625	16, 0.0625	190	158	46
	0.85	0.832 (±0.052)	0.933 (±0.041)	0.663 (±0.114)	8, 0.0625	4, 0.03125	8, 0.25	205	122	29
	0.8	0.829 (±0.077)	0.921 (±0.044)	0.678 (±0.033)	16, 0.03125	0.5, 0.0625	4, 0.0625	171	102	48
	0.75	**0.836 (±0.051)**	0.933 (±0.041)	0.676 (±0.134)	**4, 0.03125**	2, 0.0625	32, 0.007812	172	103	71
	0.7	0.796 (±0.070)	0.933 (±0.048)	0.616 (±0.079)	8, 0.0625	4, 0.0625	16, 0.007812	150	107	63
0.15	0.9	0.798 (±0.066)	0.928 (±0.048)	0.667 (±0.121)	2, 0.125	2, 0.125	4, 0.0625	138	109	53
	0.85	0.788 (±0.069)	0.934 (±0.023)	0.663 (±0.114)	4, 0.125	2, 0.0625	8, 0.25	134	102	29
	0.8	0.776 (±0.072)	0.926 (±0.045)	0.678 (±0.033)	4, 0.125	2, 0.0625	4, 0.0625	92	80	48
	0.75	0.802 (±0.055)	0.908 (±0.046)	0.676 (±0.134)	4, 0.125	4, 0.125	32, 0.007812	114	110	71
	0.7	0.787 (±0.057)	0.922 (±0.049)	0.616 (±0.079)	2, 0.0625	0.5, 0.03125	16, 0.007812	99	82	63
0.2	0.9	0.773 (±0.072)	0.920 (±0.069)	0.736 (±0.123)	8, 0.03125	1, 0.125	4, 0.0625	70	77	48
	0.85	0.765 (±0.056)	0.908 (±0.063)	0.580 (±0.145)	8, 0.125	4, 0.125	2, 0.25	69	94	37
	0.8	0.768 (±0.028)	0.914 (±0.037)	0.638 (±0.087)	8, 0.125	0.5, 0.25	16, 0.0625	72	76	68
	0.75	0.763 (±0.069)	**0.933 (±0.041)**	0.609 (±0.115)	8, 0.03125	**2, 0.25**	4, 0.25	60	56	28
	0.7	0.774 (±0.067)	0.919 (±0.031)	0.633 (±0.113)	16, 0.125	1, 0.0625	2, 0.25	64	65	32
0.25	0.9	0.742 (±0.058)	0.921 (±0.036)	0.707 (±0.100)	4, 0.25	1, 0.25	16, 0.0625	40	63	32
	0.85	0.755 (±0.045)	0.892 (±0.066)	0.673 (±0.087)	8, 0.25	16, 0.015625	32, 0.0625	38	60	33
	0.8	0.759 (±0.064)	0.913 (±0.050)	0.669 (±0.097)	4, 0.5	2, 0.125	32, 0.0625	39	69	26
	0.75	0.760 (±0.083)	0.893 (±0.076)	**0.740 (±0.100**)	8, 0.5	0.5, 0.25	**4, 0.125**	29	41	25
	0.7	0.739 (±0.064)	0.878 (±0.051)	0.602 (±0.110)	2, 0.5	1, 0.25	8, 0.125	27	42	26
0.3	0.9	0.684 (±0.075)	0.908 (±0.053)	0.599 (±0.161)	2, 0.5	1, 0.25	16, 0.0625	22	53	31
	0.85	0.698 (±0.052)	0.906 (±0.055)	0.656 (±0.093)	1, 1	2, 0.125	16, 0.25	21	37	25
	0.8	0.698 (±0.052)	0.914 (±0.036)	0.701 (±0.131)	1, 1	1, 0.5	8, 0.25	21	38	31
	0.75	0.699 (±0.055)	0.907 (±0.064)	0.654 (±0.149)	1, 1	1, 0.5	16, 0.125	20	33	25
	0.7	0.673 (±0.064)	0.893 (±0.073)	0.573 (±0.050)	0.5, 1.0	2, 0.015625	32, 0.125	16	33	22

Table B. False positive rates (FPRs) and sensitivity values obtained by PSIONplus and BLAST on the three test datasets.

Measure	Methods	$TEST30_{ION}$	$TEST30_{VLG}$	$TEST60_{VGS}$				
				Potassium	Anion	Calcium	Sodium	average
FPR	BLAST	1	52.9	18.5	0	0	0	4.6
	PSIONplus	19.2	52.9	10.1	0	2.6	0	3.2
Sensitivity	BLAST	47.9	76.7	100	76.6	83.7	87.0	86.8
	PSIONplus	90.4	76.7	99.2	78.7	93.9	87.0	89.7
